# The role of density-dependent and –independent processes in spawning habitat selection by salmon in an Arctic riverscape

**DOI:** 10.1371/journal.pone.0177467

**Published:** 2017-05-22

**Authors:** Brock M. Huntsman, Jeffrey A. Falke, James W. Savereide, Katrina E. Bennett

**Affiliations:** 1 Institute of Arctic Biology, University of Alaska Fairbanks, Fairbanks, Alaska, United States of America; 2 U.S. Geological Survey, Alaska Cooperative Fish and Wildlife Research Unit, University of Alaska Fairbanks, Fairbanks, Alaska, United States of America; 3 Alaska Department of Fish and Game, Division of Sport Fish, Fairbanks, Alaska, United States of America; 4 Los Alamos National Laboratory, Los Alamos, New Mexico, United States of America; University of Alberta, CANADA

## Abstract

Density-dependent (DD) and density-independent (DI) habitat selection is strongly linked to a species’ evolutionary history. Determining the relative importance of each is necessary because declining populations are not always the result of altered DI mechanisms but can often be the result of DD via a reduced carrying capacity. We developed spatially and temporally explicit models throughout the Chena River, Alaska to predict important DI mechanisms that influence Chinook salmon spawning success. We used resource-selection functions to predict suitable spawning habitat based on geomorphic characteristics, a semi-distributed water-and-energy balance hydrologic model to generate stream flow metrics, and modeled stream temperature as a function of climatic variables. Spawner counts were predicted throughout the core and periphery spawning sections of the Chena River from escapement estimates (DD) and DI variables. Additionally, we used isodar analysis to identify whether spawners actively defend spawning habitat or follow an ideal free distribution along the riverscape. Aerial counts were best explained by escapement and reference to the core or periphery, while no models with DI variables were supported in the candidate set. Furthermore, isodar plots indicated habitat selection was best explained by ideal free distributions, although there was strong evidence for active defense of core spawning habitat. Our results are surprising, given salmon commonly defend spawning resources, and are likely due to competition occurring at finer spatial scales than addressed in this study.

## Introduction

Determining the relative strength of exogenous and endogenous mechanisms on population growth is important to achieve conservation goals in natural populations.

Historically, endogenous (density-dependent, DD) or exogenous (density-independent, DI) processes individually were argued to control population dynamics [1], yet current research indicates that these mechanisms act in concert. Indeed, Turchin [2] argued that “A much more fruitful approach… is to estimate the relative strengths of exogenous versus endogenous contributions to population change”. Many studies have since adopted this combined approach to explain mechanisms responsible for population dynamics [3–5]. An interesting pattern that commonly influences the relative strength of DD vs. DI control is spatial proximity to “core” habitats [6–8]. According to the abundant center hypothesis, a population should be most strongly regulated by DD processes at the core of its distribution [9,10] where population densities are high and likely less susceptible to perturbation from stochastic environmental conditions [6,11]. However, population dynamics are not exclusively limited by local demographic performance (*i*.*e*. birth and death rates), and population change via behavior (*e*.*g*. habitat selection via dispersal) should also be considered [12].

Habitat selection theory predicts that an individual should select habitats that maximize fitness [13,14]. The ideal free distribution hypothesis (IFD) states that individuals within a population have equal access to resources, resulting in reduced fitness at high densities [15]. Limited resources due to competition then forces individuals to decide whether to leave a potentially inferior habitat to maximize fitness. However, many populations demonstrate dominance hierarchies, in which resources are not equally distributed. Under these scenarios, also known as ideal pre-emptive and Ideal despotic distributions (IDD), dominant individuals defend resources and susceptibility to DD mechanisms is not equivalent for all individuals within the population [12]. Because resources are not evenly distributed among individuals, the IDD predicts that reduced resource acquisition is exacerbated for inferior competitors. Mean fitness would decrease with increasing densities under the IDD but relative differences between dominant and subordinate individuals are predicted to be greater than under the IFD. As a result, identifying landscape features for which habitat selection decisions are made can assist with determining what limits population productivity.

When habitat selection leads to maximizing fitness, the result is in an evolutionary stable strategy [16]. Morris [17] provided a novel approach to infer relative fitness differences between habitat patches by interpreting between-habitat density plots, known as isodars. The resulting isodar is therefore a solution to ideal habitat selection [16–19]. This concept has been used to explain habitat selection dynamics for numerous taxa, including feral horses, deer mice, Arctic rodents, multiple fish species, macroinvertebrates, and unicellular organisms [20–25]. Isodars have also been used to test IFD vs IDD population structuring [26], identify the presence of attractive sinks (i.e., ecological traps; [27]), estimate the number of patches within a landscape [28], and measure Allee effects [29]. Clearly, isodars can provide important insight into the habitat selection dynamics of a population and critical information valuable for species conservation.

Chinook salmon (*Oncorhynchus tshawytscha*) are an important socioeconomic and ecological resource across the Pacific Northwest of North America, but are listed as threatened or endangered throughout much of their range [30]. Multiple factors have been implicated in population declines of this species across their complex life-cycle. Following emergence from redds (nests), juvenile Chinook salmon migrate directly to sea (ocean type life-history) or spend one year rearing in freshwater (stream type life-history). After spending multiple years feeding in the marine environment, adults typically return to natal streams to spawn. This complex life-cycle exposes Chinook salmon to multiple stressors across marine and freshwater environments. For example, DI factors such as river flow and temperature affect juvenile survival in freshwater [31–33], whereas flow regimes in natal spawning streams affect spawning site selection by returning adults [23]. Interestingly, evidence during freshwater phases of Chinook salmon life-histories [34–36] suggests competition for limited resources may be an important regulating mechanism. Indeed, intraspecific competition for ideal spawning habitats is well documented in salmon [37], indicating analysis at this life-stage may provide an opportunity to test habitat selection theory (*i*.*e*. the relative strength of DD vs. DI factors) and contribute towards the conservation and management of this important species.

Located in interior Alaska, U.S.A., the Chena River historically supported the second largest escapement (number of adult salmon not captured in the fishery that return to spawn) with significant sport and subsistence Chinook salmon fisheries within the U.S.A. portion of the Yukon River basin. Similar to other populations in Alaska, the Chena population has experienced a period of low abundance in recent years [38]. Mechanisms driving these patterns are unknown, but could be due to changing environmental conditions in marine or freshwater ecosystems, or simply a result of natural population dynamics. Alaska Chinook salmon populations remain much healthier than most in the Pacific Northwest [39,40], making them an ideal population to test the significance of DD vs DI mechanisms on habitat selection. However, investigation of the relative strength of DI vs. DD across riverscapes [41] remains a challenge for Alaska salmon populations because detailed digital representations of key DI factors such as geomorphology, water temperature, and river flows are lacking for this region.

**Fig 1:**
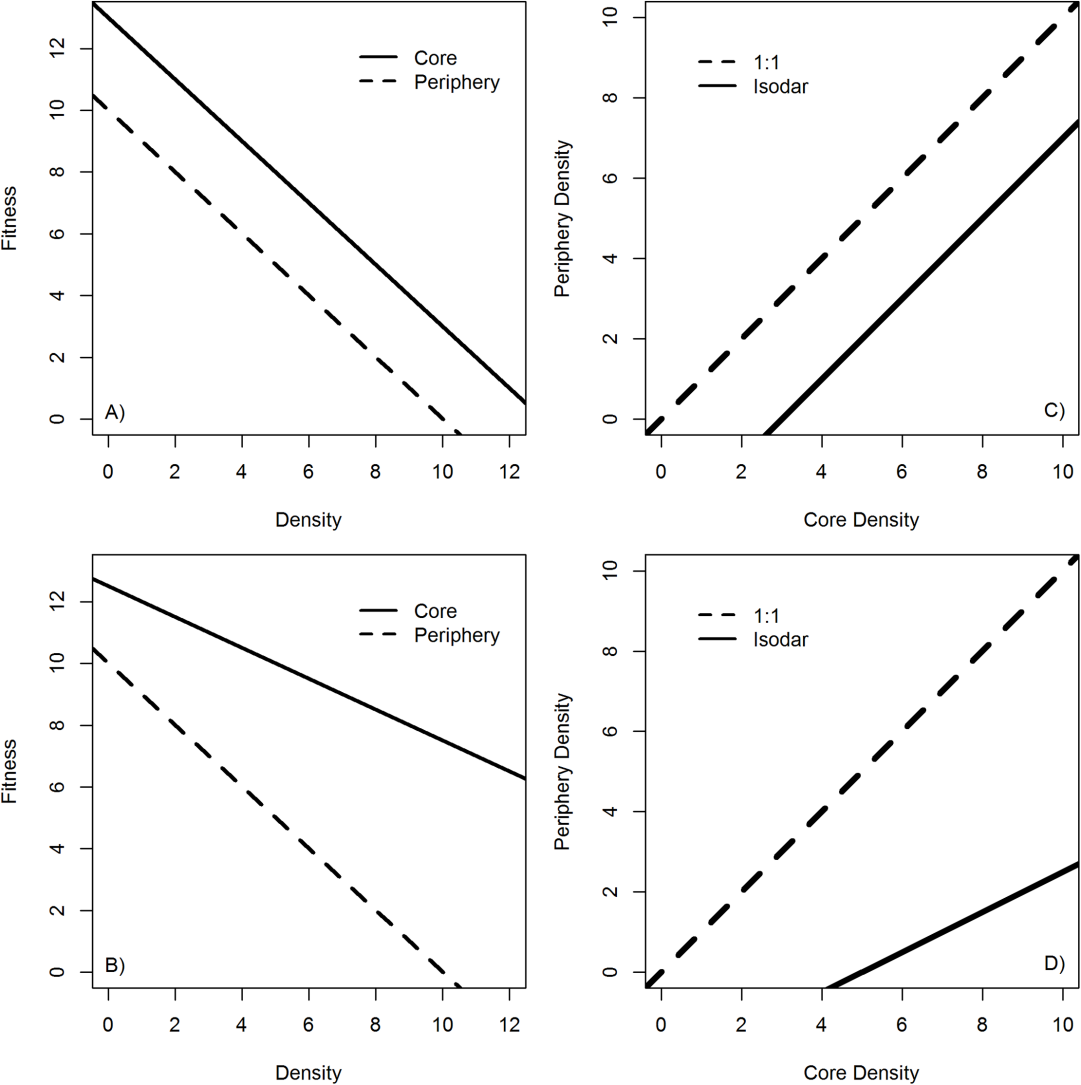
Conceptual models adapted from Morris [29] demonstrating the relationship between the fitness-density curve and isodar plots under density-dependent habitat selection. (A) Illustrates relative differences in fitness between the core and periphery and (C) the resulting isodar curve when habitat quality is not significantly different between habitats (slope = 1) under ideal free distributions (IFD). Panels (B) and (D) demonstrate relative differences in fitness between core and periphery and the resulting isodar curve when habitat quality is significantly different between habitats (slope ≠1) under IFD.

Our goal was to explore how DD and DI processes influence spatial distributions and habitat selection dynamics of spawning Chinook salmon in the Chena River. Our objectives were to, 1) develop spatially and temporally explicit models of DI variables hypothesized to influence habitat selection decisions, 2) predict spawner counts within core and periphery spawning areas based on DI and DD variables, and 3) develop isodars of core and periphery spawning habitat densities to determine relative differences in habitat quality and quantity between the two habitat types and infer whether core habitats are actively defended. We anticipated both DD and DI mechanisms to control Chinook salmon spawner counts, where the strength of DD was predicted to be strongest in the core while DI was predicted strongest in the periphery. We also predicted significantly higher quantity and quality of spawning habitat in the core than the periphery, as evaluated by isodar analysis (Fig 1). Lastly, we predicted that core spawning habitat would be aggressively defended by dominant fish as evidenced by a non-linear isodar in favor of core habitat.

## Methods

### Ethics statement

Data was collected by state biologists and made readily available to the community. Observations were made by aerial surveys, and no animals were handled.

### Study site

**Fig 2:**
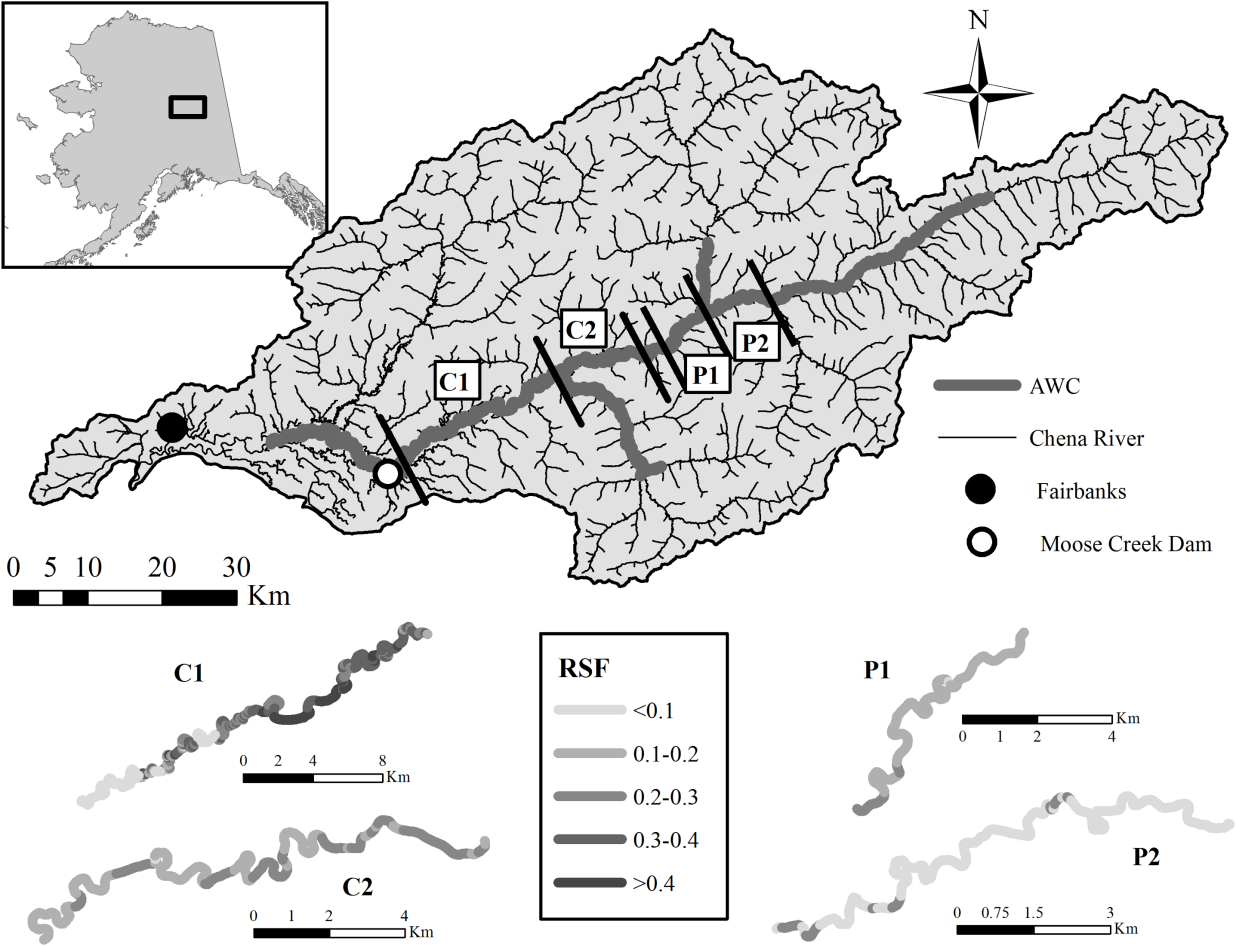
Map of the Chena River basin and its location in Alaska, USA (inset). The Core (C1 and C2) in the top panel represents the concentrated spawning area identified by aerial surveys and state biologists during carcass surveys, with the periphery (P1 and P2) located northeast of the core. The known distribution of spawning Chinook salmon in the Chena watershed is depicted by the Anadromous Waters Catalog (https://www.adfg.alaska.gov/sf/SARR/AWC/). The bottom panel shows predicted spawning habitat quality (range 0–1; darker is higher quality) based on resource selection function analysis for each study reach. The Middle Fork confluence is identified by MFC.

The Chena River is a large clear water tributary (drainage area ~ 5,130km^2^) of the Tanana River, which is part of the Yukon River drainage in Alaska, U.S.A. (Fig 2). Flow within the Chena River is derived from four major tributaries and hydrology is primarily driven by snow melt, precipitation, and groundwater from unconfined aquifers [42]. Mean annual flow is 38.5 m^3^ s^-1^ over the period of record, although construction of the Moose Creek Dam has prevented flows downstream of the dam from exceeding 340 m^3^ s^-1^ since its completion in 1981.

### Aerial counts

We compiled aerial survey counts of spawning Chinook salmon conducted during 1971 to 2014 from a database managed by the Alaska Department of Fish and Game (ADFG, S1 Table). Survey conditions (good, fair, poor, or undefined) were noted during each survey, and surveys were conducted up to 7 times within a year (1992) to capture peak spawn timing. We considered the maximum count for each of the four stream study reaches as our data point for a location and year because peak counts provide the most likely scenario under which strong intraspecific competition may occur, as well as being a common response variable modelled in other aerial survey count studies [23, 43]. We reduced the number of years (*n* = 10) in which peak counts were used for count model development by only considering years in which counts were made within four spatially defined stream reaches. These sections of stream (study reaches from this point forward) were in the core (C1 and C2) and periphery (P1 and P2), and were selected because they were the most consistently flown segments (Fig 2, S1 and S2 Tables).

Count model estimates can be biased by not accounting for heterogeneity in detection efficiency across sampling locations and through time [44]. Detection efficiency of aerial surveys is difficult to measure owing to lack of survey replication and meeting necessary closure assumptions. We explored the effect of survey condition on aerial counts by comparing total aerial count peak densities (total density = summed peak counts^1^ stream km^-1^) to escapement estimates for the Chena River Chinook salmon population (Supplementary Material, S1 File -*Test description for bias in detection efficiency*). We found a strong positive relationship between escapement estimates and total peak densities (S1 Fig, *p* = 0.002, *R*^*2*^ = 0.65), as well as slightly higher predicted counts than observed counts during poor quality surveys (S2 Fig). As a result, we assumed spatial and temporal variability in aerial count detection efficiency was minimal for two reasons. First, a strong relationship between total densities and escapement suggested limited temporal variability in detection efficiency, further supported by survey condition via residual plots. Second, within-year survey conditions were consistent among study reaches with the exception of 1999 where conditions were fair for all locations except in P2 which was poor.

### Resource selection functions

We used a digital landscape model (NetMap; Earth Systems Institute, Mt. Shasta, CA) parameterized for the Chena River basin to derive hydrologic and geomorphic DI habitat attributes potentially important to spawning Chinook salmon. The NetMap model generates a synthetic digital stream network from a remotely-sensed digital elevation model (DEM) based on flow accumulation and channel delineation algorithms [44,45]. The result is a network of 20–200 m sub-reaches throughout the entire Chena River watershed (2,265 stream-km) to which physical attributes are assigned based on empirical geomorphic relationships [46]. We used three physical attributes that strongly correlated with Chinook salmon spawning habitat suitability in other regions [47,48] to predict spawning suitability for each of our four study reaches: stream gradient (GRAD; %), bankfull width (BFW; m), and valley width index (VWI; a measure of valley constraint; unitless). These three attributes showed low multicollinearity (variance inflation factor, VIF < 3 [49]) and were used to develop a resource selection function model (RSF).

Our RSF was fit using the regression approach outlined by Nielson and Sawyer [50]. The response, Chinook salmon redd counts, was estimated from independent aerial surveys (i.e., the surveys were not included in subsequent count models) conducted in 2005 and 2006 [51]. During each survey, counts of redds were georeferenced and subsequently associated with a sub-reach. We modeled redd counts as a function of GRAD, BFW, and VWI based on a negative binomial distribution using the “glm.nb” function in the R statistical program’s MASS package [52]. Median parameter estimates and 90% confidence intervals for each covariate were estimated using a bootstrapping technique [53] wherein we fit the model for 1000 permutations [54] using all 176 used sub-reaches along with an equal number of randomly selected available sub-reaches from the known spawning distribution of Chinook salmon in the basin (*n* = 2536 sub-reaches, Anadromous Waters Catalog, Fig 2). The resulting model was used to predict intensity of use (from this point forward “RSF score”) for all sub-reaches within the four study reaches by rescaling predicted counts between 0 and 1 [50]. Each of the four study reaches (C1, C2, P1, and P2; measured at a coarser spatial scale than sub-reaches for RSF analysis) was assigned the mean RSF score from all nested 50–200 m sub-reaches and used as a fixed effect DI covariate for our count model (*see below*).

### Flow model

We used a semi-distributed water-and-energy balance hydrologic model (variable infiltration capacity model, VIC; [55]) parameterized for the Chena River [56] to estimate mean daily flows (m^3^ s^-1^) for the four study reaches across the study period. Predicted flows were used because they provided unique spatial and temporal flow estimates for each study reach. We used the approach proposed by Wenger et al. [57] to downscale daily flow predictions produced by the base model to the stream reach-scale. We constructed hydrographs for each study reach using flow predictions at the farthest downstream end of each study reach. We then developed three DI flow metrics for each study reach during each year in which aerial spawner counts were available, including maximum and mean daily flow (m^3^ s^-1^), and flow variability (C.V. of mean daily flows). All metrics were developed for the interval in which spawning occurs for Chinook salmon of the Chena River (01-July to 31-Aug).

### Temperature model

Stream temperature data were collected using HOBO Pro V2 data loggers within each of the four study reaches during June 2005-November 2006 [51]. We built predictive models using multiple stepwise linear regression to predict stream temperature within each of the four study reaches during years in which aerial counts were made. Predictor variables used for model construction were extracted from a local climate station in Fairbanks, AK (wunderground.com). We removed highly correlated climate variables (Pearson’s *r* > 0.6), which resulted in five climate station variables (mean daily air temperature, maximum daily humidity, square root transformed minimum daily humidity, barometric pressure, and wind velocity) used to predict stream temperature in each study reach. Variables were sequentially removed during model development using deletion tests within a stepwise regression framework. Deletion tests remove the least significant parameter from a model and F-tests (α = 0.15) identify the most parsimonious model that balances complexity and explained variability [58]. Final models were used to backcast mean, minimum, and maximum stream temperatures based on historic climatic variables for each study reach and year combination.

### Count model

We used a generalized linear model to predict Chinook salmon peak spawner counts within study reaches (*i*.*e*. C1, C2, P1, and P2) as a function of DD and DI factors based on a negative binomial distribution. Fifteen candidate models (Table 1) were developed and ranked to determine whether DD, DI, or a combination best explained counts using an information theoretic approach and AIC model-selection [59] with the MuMin package [60] in R. A categorical spatial reference variable (reference to being in the core or periphery, CP) was also included as a fixed effect. Annual carcass surveys conducted by Alaska Fish and Game biologists have identified segments of stream between the lower end of C1 and upper end of C2 to account for the majority of Chinook salmon spawners within the watershed (J. Savereide unpublished data), and therefore aerial surveys outside of this focused length of stream was considered peripheral habitat. We identified and removed multicollinear predictor variables with high (>3) VIF’s which resulted in three DI variables included in candidate models: average flow, minimum temperature, and mean RSF. Annual estimated escapement for the Chena River basin [61] was included to represent DD factors. Models representing DD (models 1:5, Table 1) were based on multiple combinations of additive and interactive effects between escapement and a reference to core or periphery spawning habitat. Models representing DI (models 6:9) were simple models defined previously (*Resource Selection Functions*, *Flow Model*, *Temperature Model*), with one model representing additive effects of all DI parameters. The remaining six models (models 10:15) represent a combination of DD and DI effects. Models 10:12 identify potential interactive effects between DI variables and escapement, where counts were not dependent on reference to core or peripheral spawning habitat. The remaining three models allowed interactive effects between escapement and reference to the core and periphery, and also included an interaction between the core or periphery and DI variables (models 13:15, Table 1). These models were constructed to allow DD and DI factors to vary in the core or periphery separately, testing the abundant center hypothesis. We used the resulting estimates of the relationship between predictor variables and peak counts (linear, nonlinear or interactions), to determine whether habitat selection followed an IFD (linear isodar) or IDD (curved isodar, nonlinear predictor or predictor interaction; [23]). Lastly, we included an offset based on stream length in each candidate model to account for unequal lengths of each study reach.

**Table 1:** Model selection results for candidate models predicting Chinook salmon peak spawner counts in the Chena River, Alaska.

Model	Model #	Type	df	logLik	AICc	delta	weight
E+E^2^+CP	4	DD	5	-254.38	520.5	0.00	0.36
E+CP	2	DD	4	-255.72	520.6	0.05	0.35
E*CP	3	DD	5	-255.69	523.1	2.61	0.10
E*CP+E^2^	5	DD	6	-254.35	523.2	2.72	0.09
RSF*E	12	BOTH	5	-256.41	524.6	4.06	0.05
TEMP*CP + E*CP	14	BOTH	7	-254.43	526.4	5.85	0.02
RSF*CP + E*CP	15	BOTH	7	-254.90	527.3	6.78	0.01
FLOW*CP + E*CP	13	BOTH	7	-255.55	528.6	8.08	0.01
TEMP*E	11	BOTH	5	-259.85	531.5	10.95	0.00
E	1	DD	3	-263.02	532.7	12.19	0.00
RSF	8	DI	3	-263.73	534.1	13.61	0.00
FLOW*E	10	BOTH	5	-262.93	537.6	17.09	0.00
RSF+TEMP+FLOW	9	DI	5	-263.18	538.1	17.59	0.00
TEMP	6	DI	3	-268.69	544.0	23.52	0.00
FLOW	7	DI	3	-268.70	544.1	23.55	0.00

Type represents whether the model is density-dependent (DD), density-independent (DI), or a combination (BOTH). Abbreviations are as follows: E = escapement, CP = spatial reference to core or periphery, RSF = resource selection function score, TEMP = average minimum stream temperature, FLOW = average daily maximum discharge, df = degrees of freedom, logLik = log-likelihood, AIC_c_ = Akaike information criterion corrected for small sample size, ΔAIC_c_ = difference between AIC_c_ values for the best model and selected model, and *w*_*i*_ = AIC_c_ weights. Models with “+” are additive models, whereas a “*” indicates an interaction effect was included along with additive effects.

### Model validation

Count model predictive performance was assessed using the 0.632+ bootstrap method [62–64] because this approach has been found to be less biased and result in lower variability in predictions compared to other cross-validation techniques, as well as to more reliably approximate true values with small sample sizes. The four bootstrap adjusted metrics used to assess model performance in this study were Pearson’s correlation (*r*), Spearman’s correlation (*ρ*), average error (Mean_error_), and root-mean squared error (RMSE) [64,65]. Two hundred bootstrap simulations were run for each metric. We considered the best predictive model to have the highest correlation values (*r* and *ρ*) and lowest error estimates (Mean_error_ and RMSE). Predicted counts and subsequent isodar development (see below) were then conducted using the best predictive model determined from model validation. We further assessed the performance of the best predictive model by estimating the same model performance metrics, but compared predicted counts and out-of-sample counts not used in model development (S1 Table). Out-of-sample counts were collected within the same study reaches but during different years, and were excluded from model development because all four study reaches were not monitored within the same year during these surveys.

### Isodar development

Predicted counts from the best predictive model were used to construct isodars comparing differences in habitat quantity and quality between the core and periphery. Model selection and validation identified the best predictive model depended on a spatial reference to habitat within the core or the periphery, so isodars were constructed comparing general counts within the core vs. counts in the periphery (Table 1). For example, the best model indicated the only difference between counts in C1 and C2 was based solely on stream length (the offset). Therefore, the isodar was constructed from predicted counts in the core vs the periphery, where the mean length of stream from all four stream segments was used as the offset for count predictions (21.8 km). We predicted counts in each habitat type (core or periphery) for each of the 10 escapement values used for model construction. To propagate error from predicted counts into the isodar, we employed a bootstrap where we selected 10,000 random counts from both the core and the periphery. Random counts were generated from a normal distribution from the mean and standard error of predicted counts for each escapement value from the best predictive model (again generated for the core and periphery). We calculated 90% confidence intervals for the slope and intercept of the 10,000 isodars constructed from the 100,000 bootstrapped counts at each habitat type (10,000 counts generated for each of the 10 escapement values). We considered habitat quantity to differ between core and periphery if intercept confidence intervals did not overlap zero. Habitat quantity, an intercept of zero, defined by isodars would indicate the relative difference in fitness experienced by individuals within the core versus periphery as a result of DI differences between habitats (Fig 1, [16,66]). A slope significantly different than one would indicate differences in habitat quality between the core and periphery, where differences in fitness experienced by individuals within the two habitats become greater with increasing density (Fig 1, [16,66]).

## Results

Peak Chinook salmon spawner counts ranged from a low of 32 in P2 in 1990 to as high as 1797 in C1 in 1997 (S2 Table). Study reach counts tended to fluctuate synchronously; for example, the highest number of salmon were found in 1997 for all study reaches except P2, although this was its second highest count.

### Resource selection function

Estimates of regression coefficients for the RSF model (S3 Table) indicated that the intensity of use by spawning Chinook salmon was highest where the valley was unconstrained, the bankfull width wide, and gradients low (*β*_*VWI*_ = -0.008 ± 0.002 s.e.,*β*_*BFW*_ = 0.069 ± 0.005 s.e., *β*_*GRAD*_ = -0.590 ± 0.067 s.e.). Average RSF scores were higher in core relative to periphery habitats (Fig 2), and generally decreased with distance upstream. The C1 study reach had the highest mean RSF (0.259 ± 0.161 Std.) with the lowest values observed at P2 (0.079 ± 0.016 Std., S2 Table).

### Stream flow

Mean discharge from the VIC model for the spawning period ranged from 31.6 m^3^ s^-1^ to 64.3 m^3^ s^-1^, whereas average maximum discharge ranged from 155.3 m^3^ s^-1^ to 298.3 m^3^ s^-1^ (S2 Table). In 3 of 4 study reaches, the highest mean discharge occurred in 1998, while the highest discharge in C1 was in 1986. The lowest average flows for all four study reaches were observed in 1993.

### Stream temperature

Stream temperature models had strong predictive performance across all study reaches (RMSE = 1.04°C-1.35°C, S4 Table) although all models slightly under-predicted temperatures at the upper bounds of temperature regimes (S3 Fig). Unsurprisingly, the downstream-most study reach (C1) had the warmest average maximum (12.5°C), mean (9.3°C), and minimum (5.9°C) temperatures, whereas the farthest upstream study reach (P2) was coldest (S2 Table). The warmest average predicted stream temperature for each study reach occurred in 1990 (9.0–10.2°C) and the coldest in 1998 (7.7–9.0°C).

### Count model and model validation

The top four models in our candidate set accounted for 91% of model weight, and all were DD models (Table 1). The model with the highest weight was an additive model with E, CP, and E^2^ (see Table 1 for definitions, model 4, *w*_*i*_ = 0.36). The next best model also accounted for a substantial amount of model weight (model 2, *w*_*i*_ = 0.35), and was identical to the top model but did not include E^2^. The final two models in the confidence set were similar to the top two models, but included an interaction between E and CP (Table 1). Predictive performance was similar for all of the top four models, when assessed for observed data (0.632+ bootstrap) and out-of-sample data (Table 2). Model selection and model validation indicated that the best predictive model was model 2, which we used to construct and interpret isodars.

**Table 2:** Model validation from assessment metrics.

		*r*		*ρ*		Mean_error_		RMSE	
Model	Model #	Boot	OS	Boot	OS	Boot	OS	Boot	OS
E+E^2^+CP	4	0.88	0.85	0.81	0.94	-12.00	51.12	218.83	228.09
E+CP	2	0.89	0.88	0.82	0.92	-20.36	62.64	203.66	224.04
E*CP	3	0.87	0.89	0.82	0.92	13.60	59.52	235.78	214.34
E*CP+E^2^	5	0.87	0.85	0.80	0.93	-15.32	48.02	210.81	220.05

Assessments were made from 0.632+ bootstrap corrected (Boot) and out-of-sample (OS) metrics. Pearson’s (*r*), and Spearman’s (*ρ*) correlation, average error (Mean_error_), and root-mean-squared-error (RMSE) are shown. See Table 1 for parameter abbreviation definitions. Models with “+” are additive models, whereas a “*” indicates an interaction effect was included along with additive effects.

**Fig 3:**
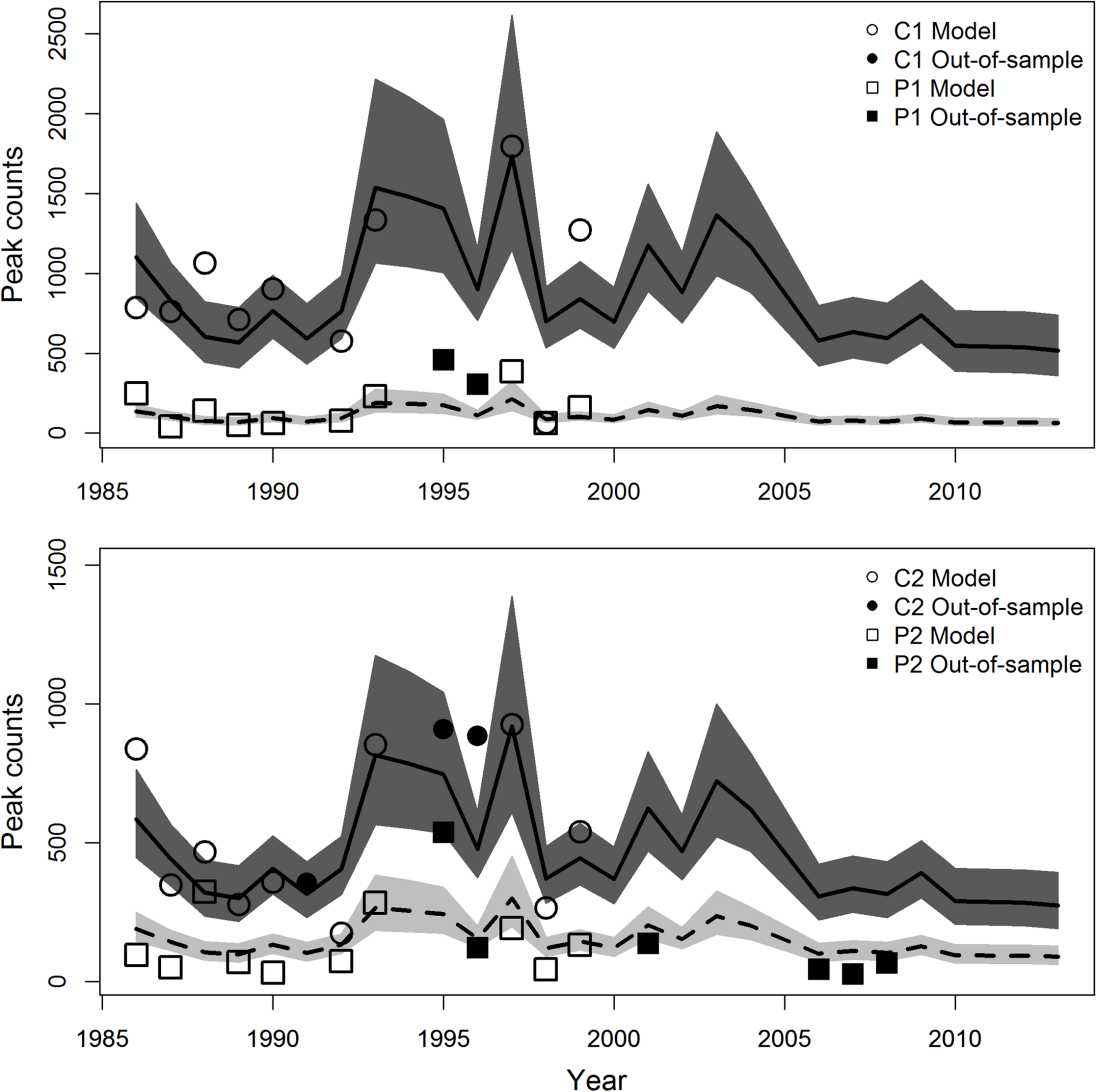
Predicted peak spawner counts from model 2 (y-axis; Table 1) as a function of annual Chinook salmon escapement estimates (1986–2013; x-axis) for the Chena River, Alaska. Observed (symbols) and predicted counts (lines and confidence ribbons) through time for C1 and P1 study reaches (top panel) and C2 and P2 study reaches (bottom panel) are shown. Dark and light grey ribbons represent 90% CIs for core and periphery habitats, respectively. Open symbols are observed counts used to fit the model and solid symbols are out-of-sample counts used for model validation. Predicted counts for each study reach are adjusted for the offset (study reach specific stream length).

Modelled peak spawner counts increased in both the core and periphery with escapement (S4 Fig). For the full time-series of escapement estimates, predicted peak spawner counts were highest in 1997 and lowest in 2013 for all study reaches (Fig 3). Consistently low counts for all study reaches were predicted from 2006 to 2013 and matched observed patterns for the Chena River basin.

### Isodar

**Fig 4:**
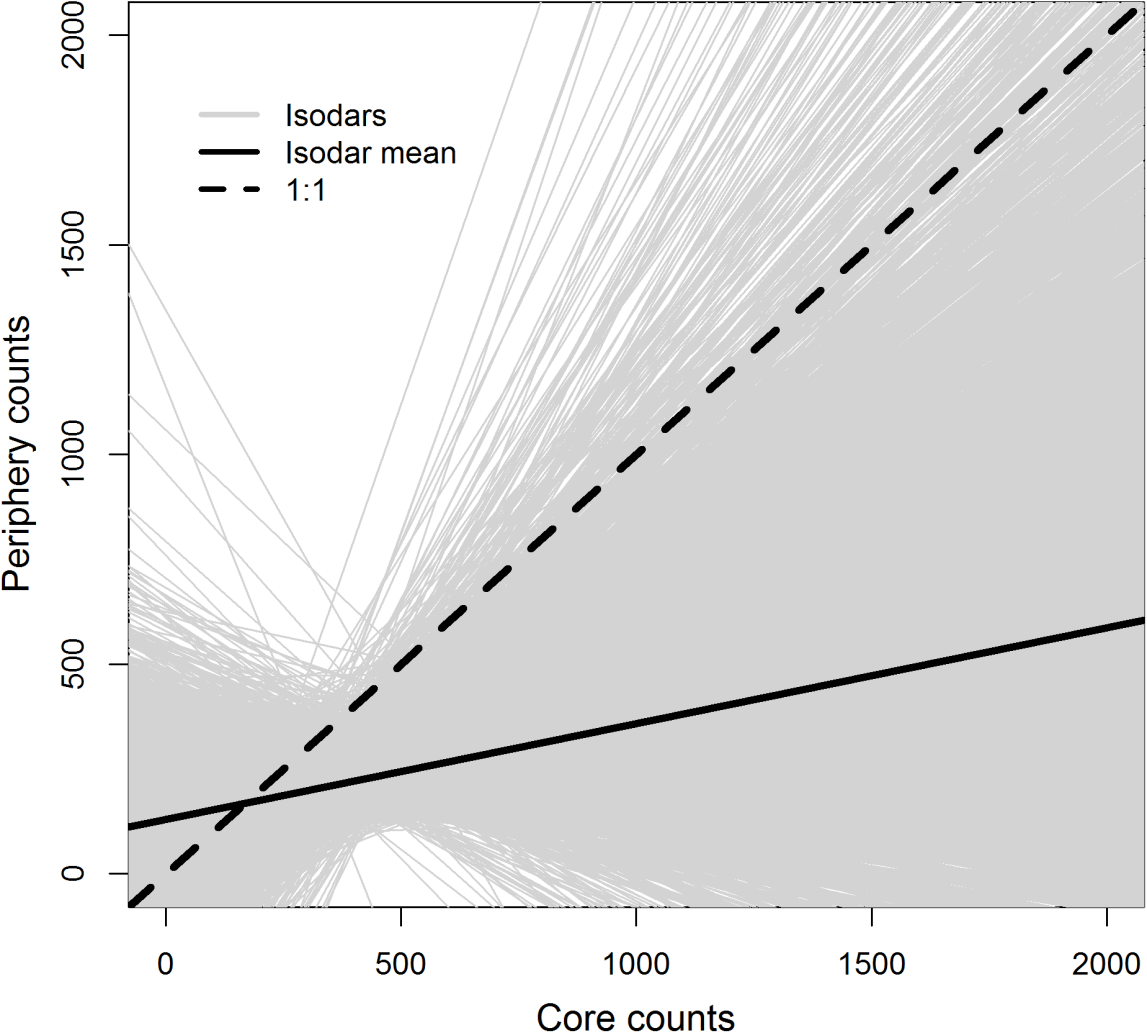
Isodars for predicted counts between core and periphery Chinook salmon spawning areas in the Chena River, Alaska based on model 2 (see Table 1 for model list) count predictions. Individual gray lines represent the 10,000 isodars developed from bootstrapped counts from both a core and periphery habitat. The solid black line represents the mean isodar from the 10,000 simulated isodars. Isodars were constructed for the core and periphery, based on count predictions from each habitat of similar length (making the offset for each habitat the mean length of all study reaches).

**Fig 5:**
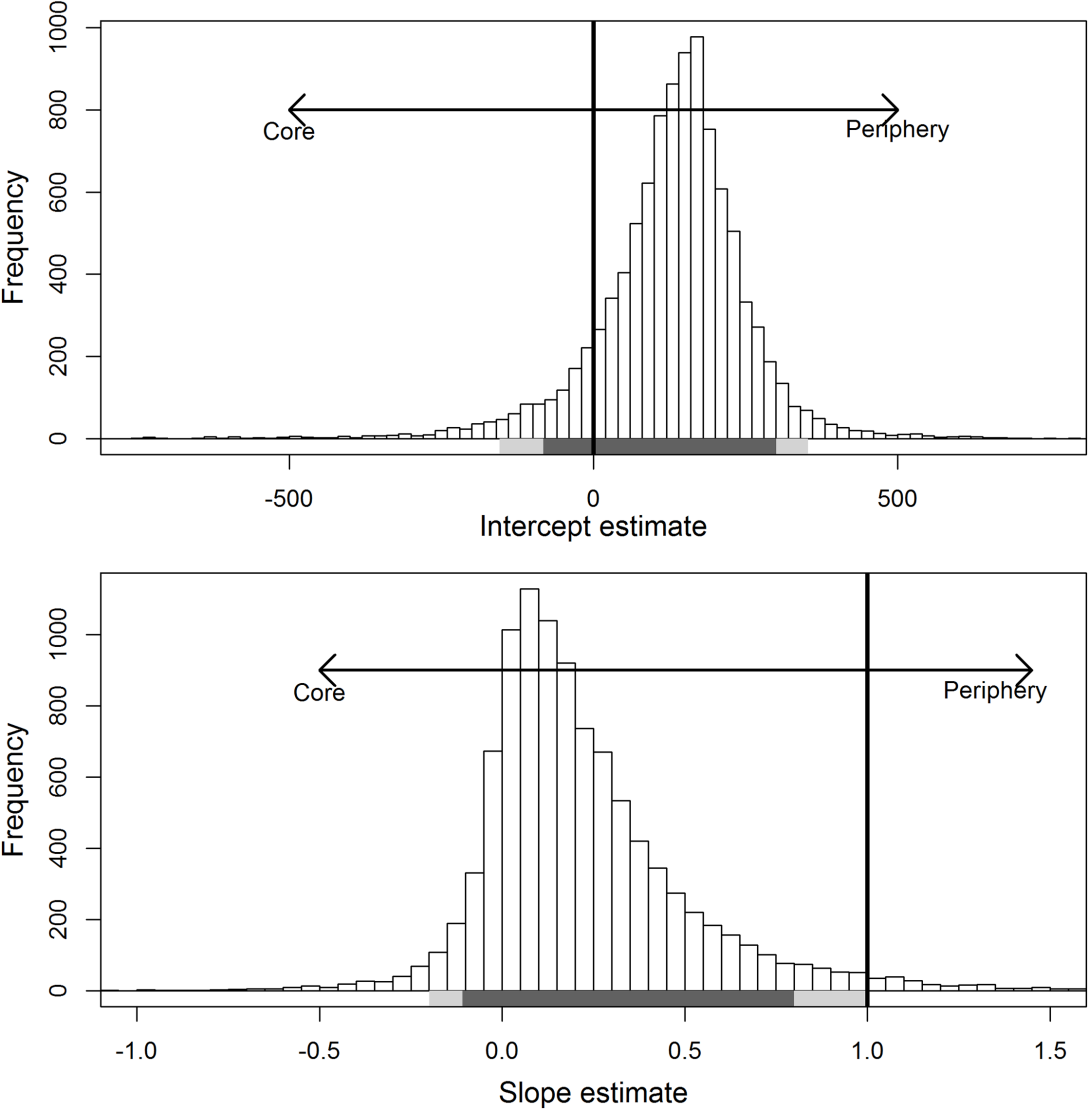
Frequency distribution of slope and intercept estimates from the isodar model comparing counts in the core and periphery. Each vertical bar indicates the frequency of the coefficient estimate (slope or intercept) from the 10,000 isodar curves. Dark vertical lines indicate the critical value in which isodar coefficients (habitat quality different = slope, habitat quantity = intercept) would favor either the core or periphery. dark gray and light gray bands below vertical bars indicate the 90% and 95% confidence intervals, respectively.

A linear isodar curve, where core Chinook salmon spawning habitats were occupied prior to peripheral habitats, was supported by the 0.632+ bootstrapped model validation which indicated model 2 was most accurate at predicting peak spawner counts. Yet, closer inspection of the regression parameters from the isodar bootstrap analysis suggested that habitat quantity was not significantly different between the core and periphery (intercept = 129.3, 90% CI = -76.4 to 297.2, Figs 4 and 5). Significant deviation of the isodar slope from a 1:1 relationship was observed (slope = 0.230, 90% CI = -0.105 to 0.780), indicating habitat quality was significantly different between the two spawning habitats based on isodar interpretation (Fig 5). Similar isodar patterns were demonstrated for isodars constructed for observed aerial counts between each core and periphery study reach (S5 Fig).

## Discussion

We took a novel approach by combining count based regression, isodar analysis, and habitat suitability modeling to identify how salmon were distributed on the riverscape and how this habitat selection behavior translated into relative fitness. Our results indicate that DD processes control spawning habitat selection in Chinook salmon of the Chena River. This adds to the growing body of evidence that adult salmon compete for high quality habitat during spawning [23,65,67,68], and the importance of inferior spawning habitat in the periphery becomes evident with saturation of the core. Although no DI variable was found responsible for habitat selection decisions, high spawning habitat suitability in the core revealed by RSF analysis showed that important spawning resources were more common in the core than the periphery of this watershed. Isodar analysis further identified the core as important Chinook salmon spawning habitat, where spawning habitat quality was particularly better in the core.

### Mechanisms distributing spawning salmon

High counts in the core spawning area were consistent with the abundant center hypothesis, where strong regulation at a population’s core is predicted to control population dynamics [10]. In our system, densities in peripheral habitats increased concurrent with overall population density suggesting strong competition for core habitats forced spawning salmon into the periphery. Periphery counts were dependent on DD processes which was further supported by the lack of evidence for any model with DI variables explaining salmon distributions at this coarse spatial scale. The prevalence of DD throughout the salmon spawning range in the Chena River provided justification for using DD habitat selection tools (*i*.*e*. isodars) to explore realized fitness advantages via habitat selection decisions.

The lack of DI factors influencing count distributions in the periphery did not conform to predictions of the abundant center hypothesis. We expected Chinook salmon spawner counts in the periphery to be more strongly influenced by DI factors such as stream temperature or flow. Chinook salmon, like many salmonids, are known to avoid habitats that exceed critical thermal ranges [69,70]. However, the Chena River is located near the northern extent of known Chinook salmon distributions and consequently supports temperature regimes (S2 Table) well below stressful thermal conditions for adult Chinook salmon (18°C, [70]). Streamflow is an equally important DI stream characteristic affecting spawning Chinook salmon habitat selection [23] and overall population dynamics have been linked to flow variability in the Pacific Northwest [33]. We did not find a significant effect of flow variability on spawner counts, which was likely due to our study reaches being relatively close in proximity to one another with low intra-annual flow variability among the study reaches. However, strong effects of flow on Chinook salmon population dynamics within the Chena River have been observed, particularly on juvenile life stages [36]. Results from that study coupled with this analysis suggest that flow variability in the Chena River has a stronger influence on juvenile salmon population dynamics than adults. Although temperature and flow are arguably the two most influential DI factors structuring aquatic populations, we found them to have relatively limited control on spawning Chinook salmon relative to DD mechanisms.

### Habitat selection within the watershed

Spawning habitat selection by Chena River Chinook salmon was better explained by IFD than a dominance hierarchy. This was surprising given what is known about aggressive defense of spawning locations by adult salmon [37,71]. However, evidence of IFD has been documented in other fish species [72,73] as well as other salmonids [20,74–76], although IDD in these studies was not directly tested. Therefore, it is not unrealistic to find evidence of IFD influencing spawning Chinook salmon distributions in the Chena River, especially at the spatial scale at which our analysis was conducted. Indeed, Morris [29] emphasized how interpretation of habitat selection can be altered simply by the spatial scale of a study. For Chinook salmon, homing can occur at spatial scales as fine as 1 km [77]. Habitats at the reach scale (our analysis; 10.6–40.5 km stream length) would be filled independent of competition, due to homing, and then competition within each reach (core or periphery) would structure how fish occupy habitats of varying quality [71]. Our RSF analysis demonstrated variability in the quality of habitat available within each study reach. Furthermore, the intercept of our isodar analysis suggested that neither the core nor periphery were more densely occupied during low escapements. This provides support for the hypothesis that Chena River Chinook salmon might home at spatial resolutions finer than the watershed scale (e.g., stream reach). However, the scale of homing by Chena River Chinook salmon is currently unknown, and further study is warranted.

Isodars indicated habitat quality was significantly better in the core than the periphery. The position of the isodar relative to the 1:1 line, being significantly lower than one, indicated the relative difference in fitness for spawning salmon between the two habitats became greater with increasing densities in both the core and periphery. If there is fine-scale homing in the Chena River Chinook salmon population, our results would suggest that greater spawning activity in the core relative to the periphery may be a result of higher demographic success of early life-history stages (*e*.*g*. egg incubation, juvenile rearing). Strong site fidelity in salmonids is common when reared in higher quality habitat (*e*.*g*. higher food productivity), while straying rates become more common when rearing habitat quality is low [48]. Intrinsic rearing habitat potential models for juvenile Chinook salmon in the Chena River predicted higher quality rearing habitat between the Moose Creek Dam and the Middle Fork confluence [78]. All core spawning habitat, as well as much of P1, is located within this same area (Fig 2). However, confirming differences in fitness between these two locations as well as fine-scale site fidelity (at least in the core) would require more research.

Alternatively, if fine-scale site fidelity was less influential in habitat selection decisions, our results would lend support to source-sink spillover effects. Density-dependent habitat selection theory has strong application to source-sink theory, in which IDD behavior leads to source-sink structuring within a metapopulation [79,12]. Increased densities in the source (core) would lead to exceeding carrying capacities and subordinate movement into lower quality sink (periphery) habitat. Spillover, or mass effects, in other fish populations [80,81], provide evidence for this hypothesis and may help explain elevated salmon densities in peripheral habitat during high escapement years. Currently, few studies have identified vital rates in Chena River Chinook salmon [36], and none to our knowledge have identified vital rates at fine spatial scales within the watershed (*e*.*g*. among stream reaches or tributaries). Quantification of egg and juvenile survival rates, as well as successful spawning dynamics (redd establishment, mate acquisition, etc.) would be required in core and periphery habitats before source-sink models could be applied. This is a dilemma throughout Alaska where knowledge of demographic rates is strongly needed during freshwater phases of salmon life-cycles [38].

Although spawner counts did not entirely conform to the abundant center hypothesis, a system that does would have unique consequences for the resulting isodar. Counts in the periphery being less dependent on DD than the core would result in counts from both habitats being out of synchrony. If mean counts in both habitats are equivalent, this would result in an isodar with a large range of potential intercept and slope estimates. The more out of phase counts in both habitats become, the more negative an isodar slope and positive an intercept. However, the abundant center hypothesis also predicts that counts should be much greater in the core than the periphery, therefore the resulting isodar would be similar to our current isodar. Specifically, the resulting isodar would demonstrate a horizontal line with a slope closer to 0 than 1. This scenario would then lead one to ask, would an isodar be appropriate to infer the relationship between habitat selection and fitness among habitats when DD does not influence habitat selection in all habitats? A similar question has been asked by Halliday and Blouin-Demers [82] when exploring the importance of DI mechanisms on habitat selection by ectotherms. Regardless, we agree with Halliday and Blouin-Demers [82] that this is an interesting avenue of future research.

## Conclusions

The role of extrinsic and intrinsic mechanisms in affecting population dynamics has been and remains a topic of much research in population biology. We found that although DI variables (*i*.*e*. habitat suitability) differed among core and periphery spawning habitats, DD processes most strongly explained habitat selection dynamics of Chinook salmon within the Chena River watershed. However, the scale at which analyses are conducted must be considered when research goals are to identify the relative strength of DD or DI in controlling population dynamics. Indeed, Westley et al. [83] revealed the strength of both intrinsic and extrinsic factors depends on spatial scale when considering Chinook salmon population dynamics (specifically reaction norms of dispersal). Such considerations should also be taken into account when testing habitat selection dynamics [29], especially for a species that must weigh habitat selection decisions with site fidelity [84]. Our results indicate that spawning Chinook salmon habitat selection is strongly dependent on DD processes, where competition for limited fine-scale suitable spawning habitat likely sets salmon carrying capacities. With accelerated degradation of important Chinook salmon habitat through climate change and anthropogenic activities [85], determining the extent to which carrying capacities may decrease will be crucial for effective conservation planning of salmonid populations.

## Supporting information

S1 Fig Regression analysis of total aerial count densities as a function of escapement for Chinook salmon of the Chena River, Alaska. Escapement values were estimated from either capture-mark-recapture (CMR) analysis or counting tower (Tower) surveys. The quality of aerial survey conditions is represented by different symbols. The “Und” indicates the survey condition was undefined.
(TIFF)

S2 Fig Boxplot of survey condition residuals from detection efficiency regression analysis in S1 Fig. The number of surveys meeting each condition is provided (*n*).
(TIFF)

S3 Fig Mean daily observed vs predicted temperature for the four study reaches. Root-mean-squared-error is represented by RMSE.
(TIFF)

S4 Fig Predicted peak counts of spawning Chinook salmon for core and periphery habitats in the Chena River, Alaska. Counts were modeled as a function of escapement (total number of spawning fish returning to the basin). Gray ribbons represent 90% confidence intervals representing significant differences in peak counts between the core and periphery.
(TIFF)

S5 Fig Isodar plot from observed Chinook salmon peak counts in the four study reaches of the Chena River, Alaska. Raw observed counts were converted to density by dividing by stream length (km). Isodars were only constructed comparing each core habitat with each periphery habitat, not within habitat type (*i*.*e*. C1-C2 and P1-P2).
(TIFF)

S1 Table Summary information on all data types used for analyses in this study. (DOCX)

S2 Table Summary statistics for abundance, habitat, temperature, and flow data for each of the four Chinook salmon study reaches in the Chena River, Alaska. (DOCX)

S3 Table Resource selection modeling results for Chinook salmon redd counts from the Chena River, Alaska. (DOCX)

S4 Table Stream temperature model coefficients and regression statistics from stepwise deletion tests reported for each study reach. (DOCX)

S1 File Test description for bias in detection efficiency. (DOCX)
