# Supplementary figures and images for: The role of density-dependent and –independent processes in spawning habitat selection by salmon in an Arctic riverscape
Source: PLoS One. 2017 May 22;12(5):e0177467. doi: 10.1371/journal.pone.0177467 (PMC5439693; doi:10.1371/journal.pone.0177467)

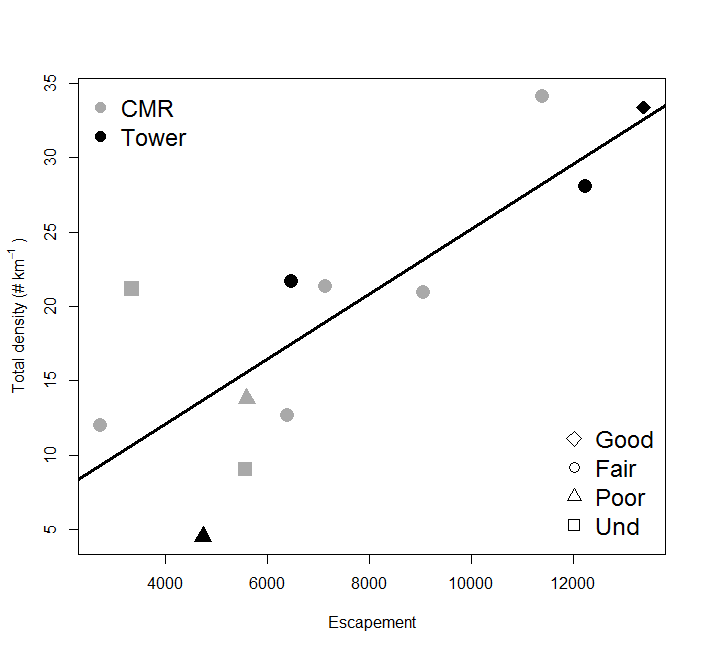

Supplement: S1 Fig — Escapement values were estimated from either capture-mark-recapture (CMR) analysis or counting tower (Tower) surveys. The quality of aerial survey conditions is represented by different symbols. The “Und” indicates the survey condition was undefined. (TIFF) [file pone.0177467.s001.tiff]

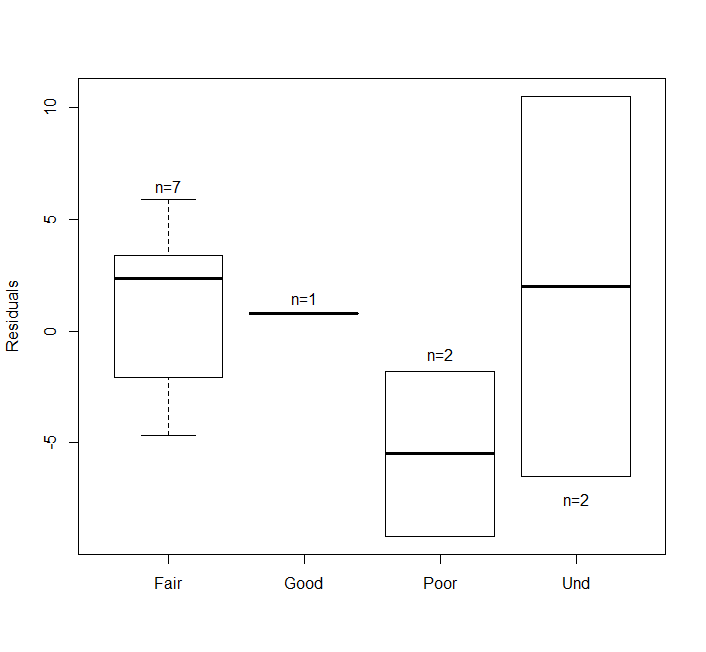

Supplement: S2 Fig — The number of surveys meeting each condition is provided (n). (TIFF) [file pone.0177467.s002.tiff]

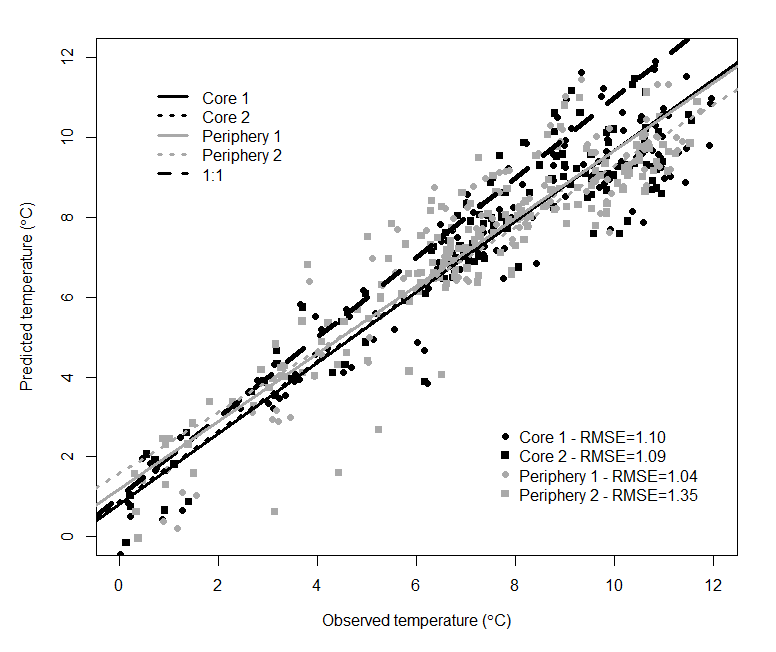

Supplement: S3 Fig — Root-mean-squared-error is represented by RMSE. (TIFF) [file pone.0177467.s003.tiff]

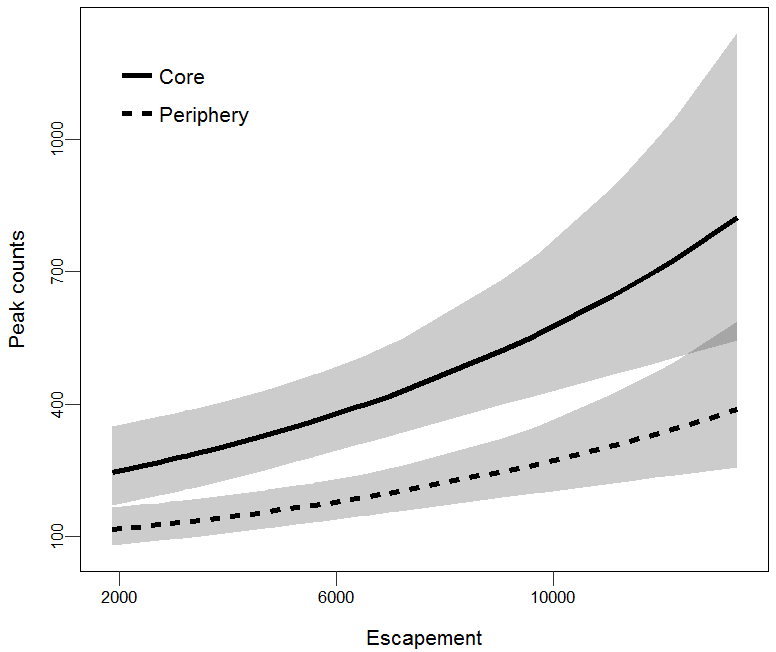

Supplement: S4 Fig — Counts were modeled as a function of escapement (total number of spawning fish returning to the basin). Gray ribbons represent 90% confidence intervals representing significant differences in peak counts between the core and periphery. (TIFF) [file pone.0177467.s004.tiff]

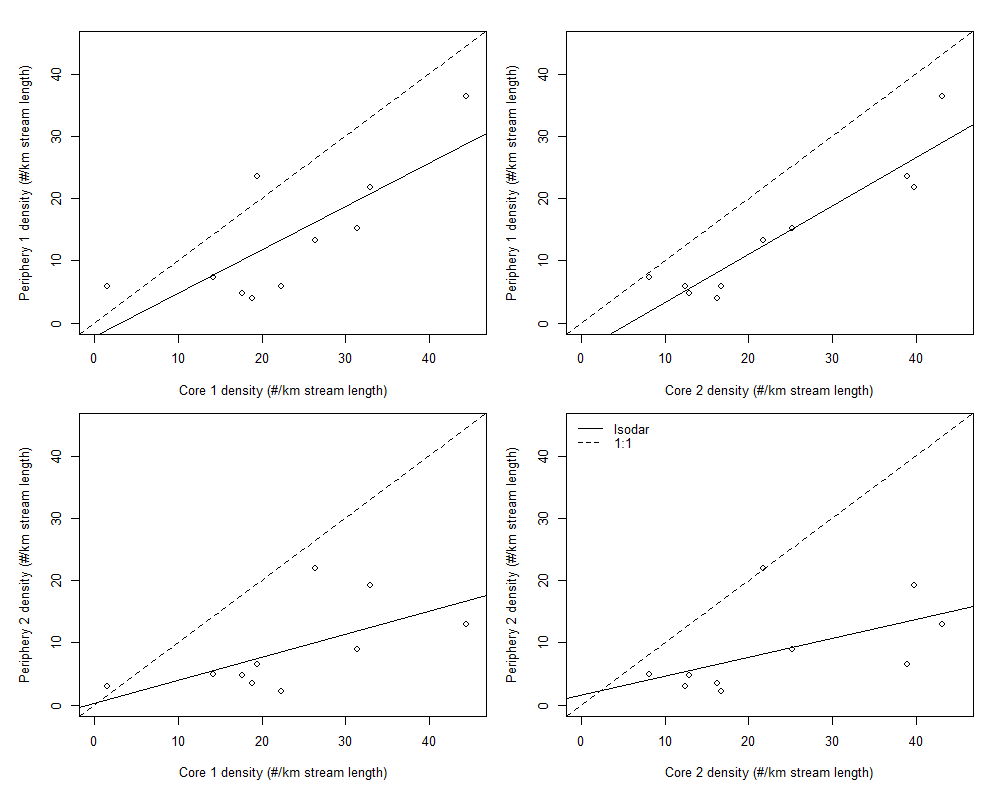

Supplement: S5 Fig — Raw observed counts were converted to density by dividing by stream length (km). Isodars were only constructed comparing each core habitat with each periphery habitat, not within habitat type (i.e. C1-C2 and P1-P2). (TIFF) [file pone.0177467.s005.tiff]
