# Supplementary material for: The role of density-dependent and –independent processes in spawning habitat selection by salmon in an Arctic riverscape
Source: PLoS One. 2017 May 22;12(5):e0177467. doi: 10.1371/journal.pone.0177467 (PMC5439693; doi:10.1371/journal.pone.0177467)
Supplement: S1 Table — (DOCX) [file pone.0177467.s006.docx]

**Supporting Information: S1 Table**

The Role of Density-Dependent and –Independent Processes in Spawning Habitat Selection by Salmon in an Arctic Riverscape

Brock M. Huntsman^1,5#^*, Jeffrey A. Falke^2#^, James W. Savereide^3+^, and Katrina E. Bennett^4+^

^1^Institute of Arctic Biology, University of Alaska Fairbanks, Fairbanks, Alaska, United States of America

^2^U.S. Geological Survey, Alaska Cooperative Fish and Wildlife Research Unit, University of Alaska Fairbanks, Fairbanks, Alaska, United States of America

^3^Alaska Department of Fish and Game, Division of Sport Fish, Fairbanks, Alaska, United States of America

^4^Los Alamos National Laboratory, Los Alamos, New Mexico, United States of America

^5^Current Address: Department of Fish, Wildlife and Conservation Ecology, New Mexico State University, Las Cruces, New Mexico, United States of America

*Corresponding author:

e-mail: [brockhunts@gmail.com](mailto:brockhunts@gmail.com)

ORCID ID: 0000-0003-4090-1949

**S1 Table.** **Summary information on all data types used for analyses in this study.**

| Analysis | Datum Type | Years Available | Location |
| --- | --- | --- | --- |
| RSF | Aerial Redd Surveys | 2005-2006 | Throughout Chena Main stem and Middle Fork |
| Count Model Development | Aerial Peak Spawner Counts | 1986-1990, 1992-1993, 1997-1999 | All Four Study Reaches |
| Out-of-Sample Prediction | Aerial Peak Spawner Counts | 1991^2^, 1995^234^, 1996^234^, 2001^4^, 2006^4^, 2007^4^, 2008^4^ | C1^1^, C2^2^, P1^3^, P2^4^ |
| Both Model Development and Validation | Escapement | 1986-2014 | Moose Creek Dam |
| Detection Efficiency Validation | Aerial Peak Spawner Counts | 1986-1990, 1992-1993, 1995-1999 | All Four Study Reaches, From Dam to C1, and From C2 to P1. |

Superscripts for out-of-sample prediction indicate years in which data was available in a particular study reach, but not used during count model development. Datum type refers to the specific type of datum used for the analysis. All aerial peak spawner counts data were collected from the Arctic-Yukon-Kuskokwim database managed by the Alaska Department of Fish and Game (<http://www.adfg.alaska.gov/CommFishR3/WebSite/AYKDBMSWebsite/Default.aspx>).
