# Supplementary material for: The role of density-dependent and –independent processes in spawning habitat selection by salmon in an Arctic riverscape
Source: PLoS One. 2017 May 22;12(5):e0177467. doi: 10.1371/journal.pone.0177467 (PMC5439693; doi:10.1371/journal.pone.0177467)
Supplement: S2 Table — (DOCX) [file pone.0177467.s007.docx]

**Supporting Information: S2 Table**

The Role of Density-Dependent and –Independent Processes in Spawning Habitat Selection by Salmon in an Arctic Riverscape

Brock M. Huntsman^1,5#^*, Jeffrey A. Falke^2#^, James W. Savereide^3+^, and Katrina E. Bennett^4+^

^1^Institute of Arctic Biology, University of Alaska Fairbanks, Fairbanks, Alaska, United States of America

^2^U.S. Geological Survey, Alaska Cooperative Fish and Wildlife Research Unit, University of Alaska Fairbanks, Fairbanks, Alaska, United States of America

^3^Alaska Department of Fish and Game, Division of Sport Fish, Fairbanks, Alaska, United States of America

^4^Los Alamos National Laboratory, Los Alamos, New Mexico, United States of America

^5^Current Address: Department of Fish, Wildlife and Conservation Ecology, New Mexico State University, Las Cruces, New Mexico, United States of America

*Corresponding author:

e-mail: [brockhunts@gmail.com](mailto:brockhunts@gmail.com)

ORCID ID: 0000-0003-4090-1949

**S2 Table.** **Summary statistics for abundance, habitat, temperature, and flow data for each of the four Chinook salmon study reaches in the Chena River, Alaska.**

|  | C1 | C2 | P1 | P2 |
| --- | --- | --- | --- | --- |
| Mean Counts (Std.) | 927.3 (474.4) | 504.7 (273.8) | 145.9 (112.8) | 129.5 (103.2) |
| Max Peak Spawner Counts ***(***Year***)*** | 1797 (1997) | 925 (1997) | 386 (1997) | 323 (1988) |
| Min Peak Spawner Counts (Year) | 63 (1998) | 174 (1992) | 41 (1987) | 32 (1990) |
| Mean Predicted Temp (°C, Std.) | 9.34 (0.42) | 9.34 (0.39) | 8.59 (0.48) | 8.45 (0.34) |
| Max Predicted Temp (°C, Std.) | 12.45 (0.42) | 11.98 (0.41) | 11.10 (0.61) | 10.66 (0.39) |
| Min Predicted Temp (°C, Std.) | 5.92 (1.47) | 5.81 (1.42) | 5.34 (0.91) | 5.57 (1.09) |
| Mean Daily Discharge (m^3^ s^-1^, Std.) | 64.3 (27.6) | 47.8 (19.0) | 45.8 (18.0) | 31.6 (12.7) |
| Max Daily Discharge (m^3^ s^-1^, Std.) | 298.3 (246.5) | 225.0 (167.0) | 217.6 (159.8) | 155.3 (108.2) |
| Average RSF (re-scaled, Std.) | 0.259 (0.161) | 0.193 (0.035) | 0.160 (0.031) | 0.079 (0.016) |
| Reach length (km) | 40.5 | 21.5 | 10.6 | 14.7 |

Values are summarized over the 10 years in which count models were constructed. Mean Counts represent the Mean Peak Spawner Counts. All temperature and discharge values are from predicted models during the summer interval (July-August). The standard deviation (Std.) is reported in parentheses, except for maximum and minimum peak spawner counts, where the year the observation was made is reported. Averages and standard deviations reported for RSF (resource selection function) were calculated by taking means over space (all stream segments within the study reach), while all other averages and standard deviations were calculated over time.
