# Supplementary material for: The role of density-dependent and –independent processes in spawning habitat selection by salmon in an Arctic riverscape
Source: PLoS One. 2017 May 22;12(5):e0177467. doi: 10.1371/journal.pone.0177467 (PMC5439693; doi:10.1371/journal.pone.0177467)
Supplement: S3 Table — (DOCX) [file pone.0177467.s008.docx]

**Supporting Information: S3 Table**

The Role of Density-Dependent and –Independent Processes in Spawning Habitat Selection by Salmon in an Arctic Riverscape

Brock M. Huntsman^1,5#^*, Jeffrey A. Falke^2#^, James W. Savereide^3+^, and Katrina E. Bennett^4+^

^1^Institute of Arctic Biology, University of Alaska Fairbanks, Fairbanks, Alaska, United States of America

^2^U.S. Geological Survey, Alaska Cooperative Fish and Wildlife Research Unit, University of Alaska Fairbanks, Fairbanks, Alaska, United States of America

^3^Alaska Department of Fish and Game, Division of Sport Fish, Fairbanks, Alaska, United States of America

^4^Los Alamos National Laboratory, Los Alamos, New Mexico, United States of America

^5^Current Address: Department of Fish, Wildlife and Conservation Ecology, New Mexico State University, Las Cruces, New Mexico, United States of America

*Corresponding author:

e-mail: [brockhunts@gmail.com](mailto:brockhunts@gmail.com)

ORCID ID: 0000-0003-4090-1949

**S3 Table. Resource selection modeling results for Chinook salmon redd counts from the Chena River, Alaska.**

|  | Estimate | SE | Low 90% CI | Up 90% CI |
| --- | --- | --- | --- | --- |
| Intercept | -8.988 | 0.219 | -9.251 | -8.682 |
| Gradient   (%) | -0.590 | 0.067 | -0.665 | -0.499 |
| Bankfull Width (m) | 0.069 | 0.005 | 0.063 | 0.075 |
| Valley Width Index | -0.008 | 0.002 | -0.011 | -0.006 |
| RMSE | 4.052 | 0.037 | 4.009 | 4.104 |

Coefficients, standard errors (SE), and upper and lower 90% confidence intervals were derived via maximum likelihood and bootstrapping. Root-mean-squared-error (RMSE) of counts with SE and 90% confidence intervals (CI) are also shown.
