# Supplementary material for: The role of density-dependent and –independent processes in spawning habitat selection by salmon in an Arctic riverscape
Source: PLoS One. 2017 May 22;12(5):e0177467. doi: 10.1371/journal.pone.0177467 (PMC5439693; doi:10.1371/journal.pone.0177467)
Supplement: S4 Table — (DOCX) [file pone.0177467.s009.docx]

**Supporting Information: S4 Table**

The Role of Density-Dependent and –Independent Processes in Spawning Habitat Selection by Salmon in an Arctic Riverscape

Brock M. Huntsman^1,5#^*, Jeffrey A. Falke^2#^, James W. Savereide^3+^, and Katrina E. Bennett^4+^

^1^Institute of Arctic Biology, University of Alaska Fairbanks, Fairbanks, Alaska, United States of America

^2^U.S. Geological Survey, Alaska Cooperative Fish and Wildlife Research Unit, University of Alaska Fairbanks, Fairbanks, Alaska, United States of America

^3^Alaska Department of Fish and Game, Division of Sport Fish, Fairbanks, Alaska, United States of America

^4^Los Alamos National Laboratory, Los Alamos, New Mexico, United States of America

^5^Current Address: Department of Fish, Wildlife and Conservation Ecology, New Mexico State University, Las Cruces, New Mexico, United States of America

*Corresponding author:

e-mail: [brockhunts@gmail.com](mailto:brockhunts@gmail.com)

ORCID ID: 0000-0003-4090-1949

**S4 Table.** **Stream temperature model coefficients and regression statistics from stepwise deletion tests reported for each study reach.**

| Variables | C1 | C2 | P1 | P2 |
| --- | --- | --- | --- | --- |
| Intercept | -4.36 | -4.25 | -43.30 | -1.78 |
| Mean Air Temp. (ºC) | 0.52 | 0.49 | 0.42 | 0.40 |
| Max Humidity (%) | 0.05 | 0.05 | - | 0.02 |
| Min Humidity (%) | 0.26 | 0.27 | 0.38 | 0.28 |
| Barometric Pressure (in) | - | - | 1.45 | - |
| Wind Velocity (mph) | - | - | -0.11 | - |
| *R^2^* | 0.89 | 0.88 | 0.85 | 0.77 |
| *p-value* | < 0.001 | < 0.001 | < 0.001 | < 0.001 |
| RMSE (ºC) | 1.10 | 1.09 | 1.04 | 1.35 |

Root-mean-squared-error of temperature is abbreviated as RMSE.
