# Supplementary material for: The role of density-dependent and –independent processes in spawning habitat selection by salmon in an Arctic riverscape
Source: PLoS One. 2017 May 22;12(5):e0177467. doi: 10.1371/journal.pone.0177467 (PMC5439693; doi:10.1371/journal.pone.0177467)
Supplement: S1 File — (DOCX) [file pone.0177467.s010.docx]

**Supporting Information: S1 Test Description**

The Role of Density-Dependent and –Independent Processes in Spawning Habitat Selection by Salmon in an Arctic Riverscape

Brock M. Huntsman^1,5^*, Jeffrey A. Falke^2^, James W. Savereide^3^, and Katrina E. Bennett^4^

^1^Institute of Arctic Biology, University of Alaska Fairbanks, Fairbanks, Alaska, United States of America

^2^U.S. Geological Survey, Alaska Cooperative Fish and Wildlife Research Unit, University of Alaska Fairbanks, Fairbanks, Alaska, United States of America

^3^Alaska Department of Fish and Game, Division of Sport Fish, Fairbanks, Alaska, United States of America

^4^Los Alamos National Laboratory, Los Alamos, New Mexico, United States of America

^5^Current Address: Department of Fish, Wildlife and Conservation Ecology, New Mexico State University, Las Cruces, New Mexico, United States of America

*Corresponding author:

e-mail: [brockhunts@gmail.com](mailto:brockhunts@gmail.com)

ORCID ID: 0000-0003-4090-1949

**S1. Test description for bias in detection efficiency.** We explored the effect of survey condition on aerial counts by comparing two independent survey approaches: 1) escapement estimates derived from capture-recapture, and 2) annual counting tower surveys (Savereide and Huang 2014). Peak counts were summed for all aerial survey locations and converted to density (total density = summed peak counts^1^ stream km^-1^). We supplemented the ten years of available aerial survey data from our four study reaches with two additional years (1995 and 1996) and locations. The additional locations were between the Moose Creek Dam and C1, and between C2 and P1 (Fig 2). These additional locations and years were excluded from count modeling, but included for detection efficiency analysis because surveys from these two locations, during the additional years, were merged with other study reaches. For example, surveys in both 1995 and 1996 reported one aerial count from the Moose Creek Dam to the end of C2. These twelve years of aerial surveys were used to check for temporal variation in detection efficiency, as our response variable for this analysis was the summed densities across all locations. We found a strong positive relationship between escapement estimates and total peak densities (S1 Fig, *p* = 0.002, *R^2^* = 0.65), as well as a slightly over-predicted relationship between peak densities from poor quality surveys and escapement estimates after inspecting residual plots (S2 Fig). Few aerial surveys fit a poor classification (*n* = 2), of which only one showed large deviation from the regression model (S1 and S2 Figs).
